# Epidemiology and outcomes of gastrointestinal mucosal melanomas: a national database analysis

**DOI:** 10.1186/s12876-022-02254-5

**Published:** 2022-04-09

**Authors:** Niraj James Shah, Mark M. Aloysius, Eldrin Bhanat, Shweta Gupta, Ganesh Aswath, Savio John, Shou-Jiang Tang, Hemant Goyal

**Affiliations:** 1 Department of Medicine, Division of Digestive Diseases, University of Mississippi Medical Center, 2500 North State Street, Jackson, MS 39216 USA; 2 Department of Internal Medicine, The Wright Center for Graduate Medical Education, 501 S. Washington Avenue, Scranton, PA 18505 USA; 3 Geisinger Commonwealth School of Medicine, 525, Pine Street, Scranton, PA 18510 USA; 4 Mississippi Colorectal Cancer Roundtable, University of Mississippi Medical Center, 2500 North State Street, Jackson, MS 39216 USA; 5 Division of Hematology-Oncology, Jr Hospital of Cook County, 1950 W Polk St, Chicago, IL 60612 USA; 6 Division of Gastroenterology, Upstate University Hospital, 750 East Adams Street, Syracuse, NY 13210 USA; 7 Division Chief Gastroenterology, Upstate University Hospital, 750 East Adams Street, Syracuse, NY 13210 USA; 8 The Wright Center for Graduate Medical Education, 501 S. Washington Avenue, Scranton, PA 18503 USA; 9 Mercer University School of Medicine, Macon, GA 31207 USA

**Keywords:** Primary mucosal melanoma, Gastrointestinal mucosal melanoma, Survival outcomes, Surgery, Chemotherapy, Radiation therapy

## Abstract

**Aim:**

Gastrointestinal malignant melanoma is a rare mucosal melanoma (MM). Other MM include the respiratory and the genitourinary tract. All mucosal melanomas have a poor prognosis when compared to cutaneous melanomas. Ano-rectal melanomas are by far the most common and most studied gastrointestinal MM. Large-scale clinical data is lacking due to the rarity of the disease. We aim to analyze epidemiology and survival of the Gastrointestinal (G.I.) MM over 45 years using a national database.

**Methods:**

The Surveillance, Epidemiology and End Results (SEER) database was queried to identify patients with biopsy-proven G.I. Melanomas. We selected tumor site, intervention, and survival information for oncology codes as per the international classification of diseases. Survival analysis was performed using the SPSS v 27 ® IBM software.

**Results:**

Of the 1105 biopsy-proven confirmed cases of primary G.I. melanoma's, 191 (17.3%) received chemotherapy (C.T.), 202 (18.3%) received radiotherapy (R.T.), 63 (5.7%) received both C.T and R.T., while 684 (61.9%) of the population received surgery alone or combined with C.T. and/or R.T. Statistically significant improvement in survival was noted in all treatment strategies that utilized surgery and also when site-specific MM cohorts underwent a surgical approach with or without C.T and/or R.T.

**Conclusion:**

This is the most extensive study reporting epidemiological and survival data of treatment strategy outcomes of primary G.I. mucosal melanoma elucidating best overall survival with a management strategy involving surgical intervention.

**Supplementary Information:**

The online version contains supplementary material available at 10.1186/s12876-022-02254-5.

## Introduction

Mucosal melanoma’s (MM) comprise approximately 1% of all melanomas [1]. It is the rarity and small number of reported cases in case series that make it difficult to stage disease. An obvious attributable risk factor (as compared to skin melanomas which are associated with ultraviolet light exposure) for the development of primary MM is lacking. Theories include migration of melanoblasts cells from the neural crest could explain its development [2].Others believe in malignant transformation of either the enteric neuroendocrine tissues of the APUD, or from neuroblastic Schwann cells of the intestinal autonomous nervous system [3–6]. Epidemiological studies have indicated a higher risk of anorectal MM in patients with HIV (Human immunodeficiency virus) infection [7–9]. MM is generally seen in older (> 70 years) non-hispanic white females, due to the involvement of the genito-urinary tract apart from other mucosal sites [1, 10, 11].

Generally, MM is more commonly seen in females with a F:M ratio of 1.8:1.0, when compared to 1:1.2 ratio seen in cutaneous melanoma[1]. Also, head and neck MM has been observed in younger population [12, 13]. G.I. mucosal melanomas are usually diagnosed late, have a more aggressive course due to the rapid lympho-vascular spread, and hence are associated with poorer outcomes [14–16]. Perineural invasion of the ano-rectal melanomas have shown to have poorer outcomes requiring an aggressive surgical approach [17]. It is estimated that up to 40% of the lesions in the GI tract could be amelanotic [14]. Experts have tried to decipher a staging system for MM, but have failed due to various differences in histology and prognostic features, and as of now it has been recommended to use the Ballantyne staging system for anorectal melanoma [14].

It has been suggested that mucosal melanocyte transformation is facilitated by a higher frequency of atypical BRAF and NRAS mutation, rendering a poorer clinical outcome for MM compared to cutaneous melanoma [18].To achieve remission, an approach of surgical resection seeking a negative margin is recommended [17, 19]. This proposed negative margin is often difficult to achieve, given the anatomical constraints of MM, along with likely multifocal lesions and robust lympho-vascular supply favoring metastasis in up to 50–90% despite an aggressive surgical approach [20].

The aggressive surgical approach has not provided a better O.S., as subsequent distant metastasis and a poor overall prognosis is the norm, and hence goals of care discussion with patient preferences and quality of life is important especially for anorectal or vulvovaginal melanoma [21, 22].Primary mucosal melanoma has a low 5-year survival of less than 25% [23]. In the recent past two decades we have seen better precision in radiotherapy delivery and several newer adjuvant chemotherapeutics in trials (Temozolomide and Nivolumab) for MM, and hope this will lead to better survival outcomes [24–26].

## Methods

### Patient selection

The National Cancer Institutes (NCI), SEER database registry from a total of 17 sites has been the pioneer for population-based cancer related survival data in the United States. It is supported by the Surveillance Research Program (SRP) in the Division of Cancer Control and population Sciences (DCCPS). We collected the de-identified data from the SEER database, a national cancer institute source for cancer incidence and survival of biopsy-proven gastrointestinal mucosal melanoma cases from 1976 till 2020 utilizing the SEER Stat (v 8.3.9 ® NCI). Survival analysis was performed using the Kaplan-Meir method, and survival distribution using Mantel-Cox chi-square test *(SPSS v 27 ® IBM). A p < 0.05 was considered to indicate a significant statistical difference. After extracting survival analysis based on overall survival, 5-year and 10-year survivals based on ethnicity, marital status, primary site of the GI melanoma and intervention (surgery, radiotherapy, chemotherapy, or any combination of the three interventions), we sub-analyzed the survival outcomes based on intervention in respective primary sites of the primary GI melanoma. Please note that we obtained the ethnicity race and origin recoded into Hispanics, Non-Hispanic American Indian/Alaska Native (NHAIAN), Non-Hispanic Asian or Pacific Islander (NHAPI), Non-Hispanic Black (NHB) and Non-Hispanic White (NHW).

## Results

### Demographics

Between 1975 and 2020, a total of 1105 biopsy-proven G.I. melanoma patients were identified and included in the study. All subjects (100%) were greater than 20 years of age. Most of the patients (83.5% of the population included) aged between 40 and 84 years. The median age was 71 years, with 63.8% of the patients diagnosed at 60 years or older. Women comprised 57.2% of the cohort, while 42.8% were men. The majority (73.8%) of the population were Non-Hispanic White, with 12.5% Hispanic, 8.1% NHAPI and 4.9% NHB patients (Table 1).

**Table 1:** Demographics with race and origin recode for MM

	Number	Percent
Hispanic (all races)	138	12.5
Non-Hispanic American Indian/Alaska Native	7	0.6
Non-Hispanic Asian or Pacific Islander	89	8.1
Non-Hispanic Black	54	4.9
Non-Hispanic Unknown Race	1	0.1
Non-Hispanic White	816	73.8
Total	1105	100.0

80.7% of all GI Melanoma cases were located either in the anal canal, rectum, or an overlapping lesion of the rectum and anal canal. 9.1% of the GI melanomas were in the stomach or esophagus, 4.7% in the small bowel and 2.6% in the large bowel. The extraluminal sites compromised of a modest 2.3% of all the GI melanomas and were located in the gall bladder, liver or pancreas. The overall survival by age was uniform over all age groups, ranging from 33.9 to 50%. 10 patients in the age group 30–34 years had the best O.S of 60%. The overall survival was not significant based on gender, though it was slightly better for males (48%) when compared to females (46.1%). The over-all survival was best for the Hispanics (all races) and NHW at 47.4% and 47% respectively, when compared to worst survival outcomes for NHB and NHAPI at 38.9% and 40.4% respectively. This difference in survival outcomes were not statistically significant with a p = 0.956 (Table 2).

**Table 2:** Overall 12-month survival based on ethnicity for MM

Race and origin recode (NHW, NHB, NHAIAN, NHAPI, Hispanic)	Total N	12-month O.S (p = 0.956)		5-year survival (%)	10-year survival (%)
		N of events	Percent		
Hispanic (all races)	137	65	47.4	33.3	33.3
NHAIAN	7	3	42.9	27.5	14.8
NHAPI	89	36	40.4	29.8	23.5
NHB	54	21	38.9	30.1	25.8
Non-hispanic unknown race	1	0	0.0	0.0	0.0
NHW	815	383	47.0	30.7	24.1
Overall		508	46.1		

N, Number; O.S, Overall survival; NHAIAN, Non-Hispanic American Indian/Alaska Native; NHAPI, Non-Hispanic Asian or Pacific Islander); NHB, Non-Hispanic Black; NHW, Non-Hispanic White

### Overall survival and cause-specific death

**Fig. 1:**
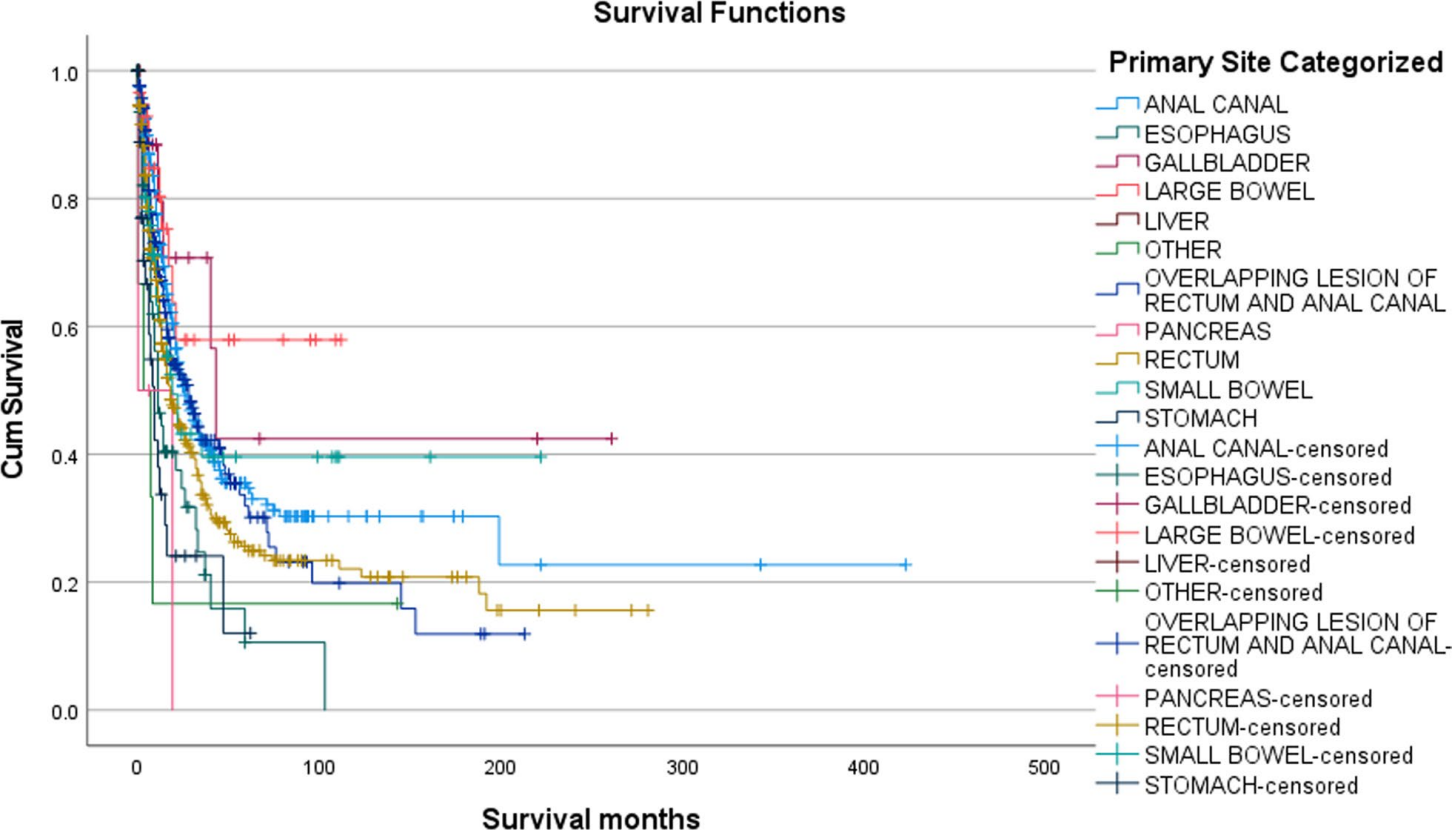
Kaplan–Meier survival curve in GI Melanoma patients based on anatomical site of the MM

The 5-year mean O.S. for the cohort for all patients with GI melanoma irrespective of site of the melanoma was 106.43 months (95% CI 89.5–123.4 months), While the median overall survival (O.S.) was 22 months (95% CI 18.7–25.2). The OS survival at 1-year, 3-year, 5-year, 10-year, and 15-years was 64.8%, 38.4%, 30.2% 25.3% and 23.1% respectively. The survival was similar for both sexes and did not differ significantly by age or ethnicity. The 1-year survival was statistically significant (p =  < 0.001) with regards to the primary site of the MM (Fig. 1). Primary pancreatic, esophageal, and gastric MM showed the worst mean O.S. (19, 23, and 36 months respectively) while small bowel, anal, anorectal, and rectal MM showed higher mean O.S. (108, 89, 65, and 63 months respectively). Case processing summary of the overall survival by primary site revealed better survival outcomes for MM of the liver (100%, only one patient), large bowel (69%), gall bladder (68.4%), when compared to poorer overall survival for MM involving the small bowel (52.9%), overlapping lesion of the rectum and anal canal (49.1%), anal canal (48%) and stomach (43.6%). The worst overall survival was noted for MM involving the esophagus (32.3% and pancreas (25%). The 10-year survival for esophageal MM and the 3-year survival for pancreatic MM was zero, while the best 15-year survival was noted for MM of the large bowel (57.9%), gall bladder (42.4%) and the small bowel (39.6%). Further analysis of the anal canal, overlapping lesion of the rectum and anal canal and rectal MM which comprised 891 (80.63%) patients, the 15-year survival was 30.3%, 11.9% and 20.8% respectively. Poorer survival in vulval MM which is likely included in the overlapping lesion of the rectum and the anal canal could be the reason why this group of patients had worse outcomes.

The cancer specific survival probability for the GI melanoma cohort was obtained from the “SEER cause-specific death classification”, as per the SEER website. "Cancer-specific Survival (CSS)" was defined as the primary melanoma-specific related death from the date of diagnosis until death. The overall SEER cause-specific death attributable to G.I. melanoma as per our analysis of the database was 54%.

### Treatment strategy and overall cancer survival

A total of 191 (17.3%) patients received chemotherapy, 202 (18.3%) received radiotherapy, while 63 (5.7%) received combined chemotherapy and radiotherapy. Of this cohort 46 (4.1%) received only chemotherapy, 30 (2.7%) received chemotherapy and radiotherapy (CRT), 82 (7.4%) received chemotherapy and underwent surgery (SC), while 33 (3%) received chemotherapy, radiotherapy and underwent surgery (SCRT). 59 (5.3%) received only radiotherapy, while 80 (7.2%) received radiotherapy and underwent surgery (SRT). 487 (44.1%) patients underwent only surgery, while 286 (25.9%) of the patients opted for, were not offered or were not candidates for any form of intervention.

The overall comparison based on treatment strategy revealed a special statistical significance of when surgery was incorporated into the plan. Patients undergoing Surgery or Surgery with chemotherapy and R.T. had better 1-year survival with statistically significant p-values of < 0.001 and 0.006 compared to other groups (Table 3). It was interesting to note that large bowel MM (N = 29) and gall bladder MM (N = 19) had the best 5-year O.S. survivals of 70% and 61.5% in the surgery group compared to 34.3% and 40% with no intervention.

**Table 3:** Treatment strategy of MM with the one-year overall survival, 5-year and 10-year survival

	N	Percent	Deaths	One year survival (%)	5-year survival (%)	10-year survival (%)
Chemotherapy	46	4.2	32	30.4	9.3	9.3
Chemotherapy + radiotherapy	30	2.7	23	23.3	12.8	12.8
No intervention	286	25.9	171	40.2	24.4	21.3
Radiotherapy	59	5.3	37	37.3	20.4	10.2
Surgery	489	44.3	220	54.8	40.0	32.4
Surgery + chemotherapy	82	7.4	56	31.7	17.3	17.3
Surgery + Chemotherapy + Radiotherapy	33	3.0	19	42.4	35.0	17.5
Surgery + Radiotherapy	80	7.2	37	53.8	30.5	30.5

*N* Number

### Patients with no intervention

The overall survival by primary site of the MM of the 286 (25.9%) patients in the NT group was statistically significant with a p = 0.28. The overall survival by primary site revealed better survival outcomes for MM of the liver (100%, only one patient), overlapping lesion of the rectum and anal canal (50%), and the stomach (47.6%), when compared to poorer overall survival for MM involving the large bowel (42.9%), rectum (41.1%), gall bladder (40%), esophagus (34.6%) and the pancreas (33.3%). The worst overall survival was noted for MM involving the small bowel (25%) and the anal canal (32.8%). While 26 (41.94%) out of the 62 patients with esophageal MM opted for NT the 15-year survival for this group was still 8.5%. Also, majority of the patients 211 (73.78%) who opted for NT had MM involvement of the anal canal, overlapping lesion of the rectum and the anal canal and the rectum. The lone patient with liver MM and 3 (75%) of patients with pancreatic MM also, opted for NT.

### Surgery and survival outcomes

**Fig. 2:**
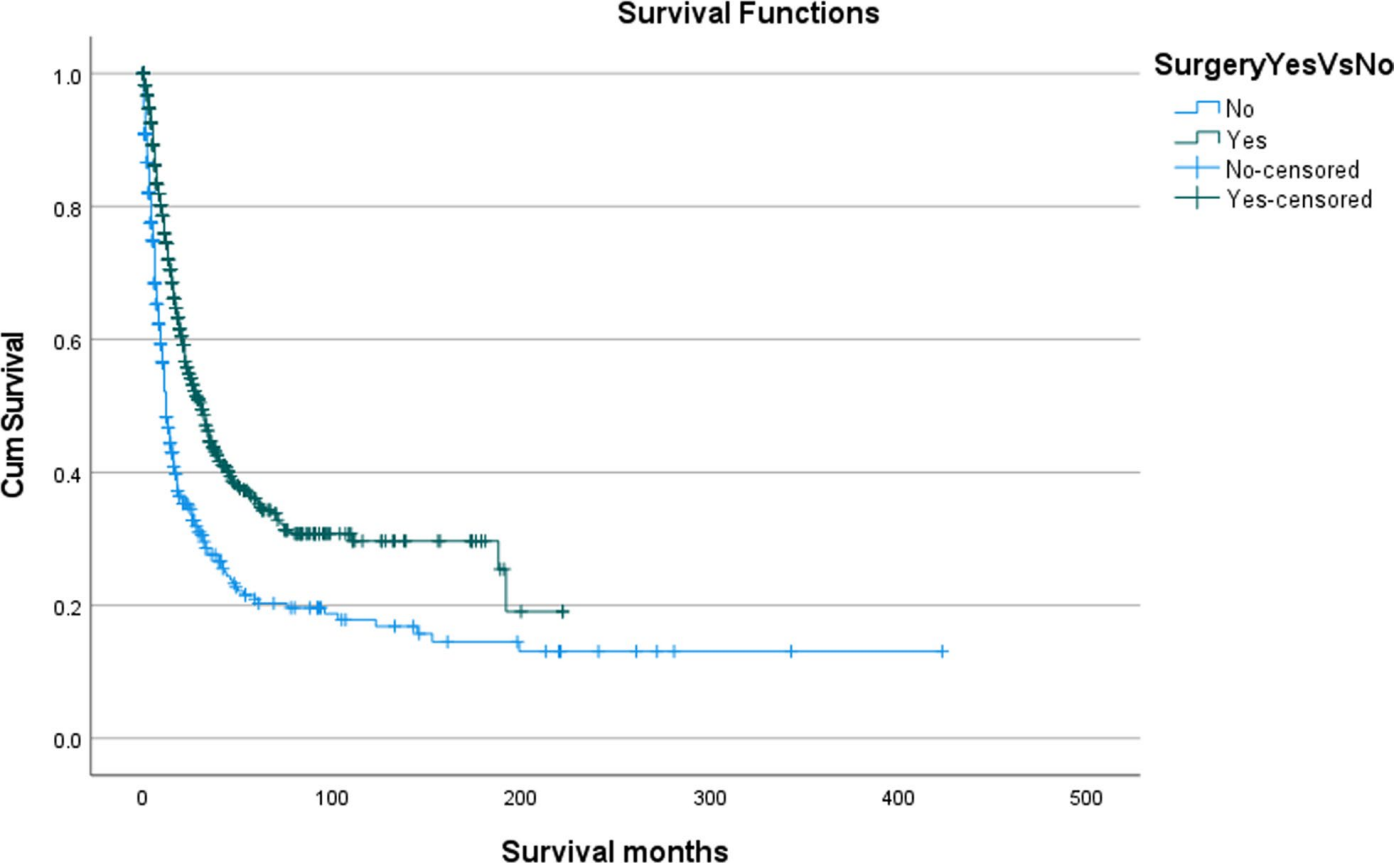
Kaplan–Meier overall survival in patients based on surgical intervention for MM

A total of 684 (61.9%) patients underwent Surgery or Surgery combined with C.T. and/or R.T. Data outcomes from the Table 2 indicates that surgery was superior, with an O.S. of 51.3%, to any other form of intervention with a significant p < 0.001 (Fig. 2).

Site-specific 5-year survival analysis of GI melanoma revealed best outcomes for patients who opted for only surgery as the intervention for the anal canal (45.5%), gall bladder (61.5%), overlapping lesion of the rectum and anal canal (33,6%), rectum (31.3%), small bowel (54%) and stomach (32.1%). Patients who had surgery only had better 5-year survival outcomes with anal canal GI melanomas when compared to surgery combined with any other modality (SC = 11.5%, SCRT 23.7% and SRT 32.6%). Also, interesting to note was that the overall survival of patients with large bowel melanoma who received SRT was 100%, compared to 34.3% with the no intervention group. (Table 4).

**Table 4:** Site specific 5-year survival when compared to treatment strategy

Primary Site	Total N	One year Survival (in %)	5-year O.S. (in %)							
			C	CRT	NT	R	S	SC	SCRT	SRT
N			46	30	286	59	487	82	33	80
p-value based onsite specific at 1 year			0.034	0.411	0.028	0.152	0.00	0.966	0.006	0.271
ANAL CANAL	325	48.0	18.5	25	21.3	50	45.5	11.5	23.7	32.6
ESOPHAGUS	62	32.3	0	0	8.5	27.7	11.6	0		
GALLBLADDER	19	68.4			40		61.5			
LARGE BOWEL	29	69.0	0		34.3		70			100
LIVER	1	100.0								
OTHER	6	16.7	0		25		0			
OVERLAPPING LESION OF RECTUM AND ANAL CANAL	214	49.1	31.3	0	40	25	33.6	34	14.3	22.2
PANCREAS	4	25.0			33.3		0			
RECTUM	352	41.8	0	11.2	26.4	0	31.3	18.5	44.4	34
SMALL BOWEL	52	51.9	50		0	0	54	41.7	50	0
STOMACH	39	43.6	0		18.6	0	32.1	50		

N, Number; O.S, Overall survival; C, Chemotherapy only; CRT, chemotherapy plus radiotherapy; NT, No therapy; R, Radiotherapy only; S, Surgery only; SC, Surgery plus chemotherapy; SCRT, Surgery plus chemotherapy plus radiotherapy; SRT, Surgery plus chemotherapy

Case processing summary of patients undergoing surgery revealed a poorer survival in patients above 65 years of age (1-year O.S. of 52.9–57.7%). Despite this difference the p-value (0.508) was not statistically different for survival outcomes based on the age of the patient.

### Chemotherapy and survival outcomes

A total of 191 (17.28%) patients underwent chemotherapy or chemotherapy combined with surgery or radiotherapy (CRT, SC, or SCRT). The O.S. outcomes were not statistically significant with age (p = 0.168), gender (p = 0.664), or site of MM (p = 0.095) in patients receiving chemotherapy. The p value based on the one-year survival was significant for chemotherapy alone (p = 0.034) and SCRT (p = 0.006), while the CRT (p = 0.411) and SC (p = 0.966) were not statistically significant.

The patients with gastric MM receiving chemotherapy had the worst outcomes with an O.S. of 16.7%. The 5-year survival was zero for patients with esophageal, large bowel, rectal and gastric GI melanoma. For small bowel GI melanoma any intervention with chemotherapy involvement produced similar 5-year survival (50% for chemotherapy alone, 41.7% for SC and 50% for SCRT). For gastric melanoma’s, addition of surgery to the regimen (SC) had better 5-year survival of 50% when compared to zero 5-year survival for chemotherapy alone. It was noted that though SCRT had the best overall one-year survival with a p = 0.006, only 17.2% of all patients who underwent chemotherapy were either offered, or opted for this regimen (SCRT). It was also noted that patients who opted no intervention had better 5-year survival than patients who opted for chemotherapy/CRT/SC/SCRT for esophageal and overlapping lesion of the rectum and anal canal melanoma at 8.5% and 40%; 5-year survival respectively (Table 4).

Case processing summary of patients undergoing chemotherapy did not reveal any particular trend in survival outcomes based on age groups when subdivided and analyzed in 5-year intervals. The p value for the overall survival was 0.168.

### Radiotherapy and survival outcomes

A total of 202 (18.28%) patients underwent radiotherapy or radiotherapy combined with surgery or chemotherapy (CRT, SCRT or SRT). The O.S. outcomes were not statistically significant with age (p = 0.413), gender (p = 0.679), or site of MM (p = 0.091) for patients receiving R.T. The patients with gastric MM who received radiotherapy had similar outcomes as patients who did not receive radiotherapy with an O.S. of 16.7%. Also, the 5-year survival of gastric MM was zero. The p value based on the one-year survival was significant for only SCRT (p = 0.006), while the radiotherapy only group (p = 0.152), CRT (p = 0.411) and SRT (p = 0.271) were not statistically significant. It was interesting to note that patients with anal canal melanomas had better 5-year survival in radiotherapy alone group (50%) as compared to any other group where radiotherapy was considered as part of the intervention (25%, 23.7% and 32.6% respectively for the CRT, SCRT or SRT groups). Also, the 5-year survival was better in radiotherapy alone group (27.7%) when compared to the CRT group where the 5-year survival was zero. For small bowel melanomas addition of chemotherapy had better 5-year survival as evident by the 50%; 5-year survival for the SCRT group compared to zero survival in the SRT group (Table 4).

Case processing summary of patients undergoing radiotherapy revealed a poorer survival in patients below 30 years of age and above 79 years of age, with these two subgroups having less than 33.3% overall survival while all the remaining age groups had > 33.3% overall survival. The p value for the overall survival based on age was not statistically significant with a p = 0.168.

## Discussion

Primary MM is a rare entity, which is challenging to diagnose, with up to 40% being amelanotic lesions. Also, one-third of the cases present with lymph node metastasis. It has an aggressive course with poor prognosis and subsequent metastasis even with a surgical approach. There are currently no established guidelines on treatment modalities for mucosal melanomas occurring in the gastrointestinal tract unlike other mucosal melanomas[26, 27]. Most published literature on gastrointestinal mucosal melanomas are from case reports and case series[2, 28–35]. A recent study from SEER database compared 872 GI MM patients to have poor survival compared with 319,327 cutaneous melanoma patients [30]. MM is poorly responsive to conventional chemo therapy and there is evidence from a pooled study for use of a PDL1 receptor antagonist (nivolumab) with a CTLA4 antagonist (ipilimumab) is synergistic and safe therapeutic strategy in MM [36].

Our study with data from the United States of 1105 subjects over the past five decades suggests surgery in any form, when incorporated into the treatment plan, proved to provide better O.S. The best 5-year survival rates were observed in patients who underwent surgery only (40%), surgery with chemotherapy and R.T. (35%), and surgery with radiotherapy (30.5%). The O.S. in the surgery sub-group also had statistically significant p-values when reviewed with specific primary tumor sites, with patients with MM of the large bowel and the gallbladder having the best 5-year survival of 70.2% and 66.9%, respectively. The overall 1-year survival for patients undergoing Surgery or Surgery with R.T. and chemotherapy had the best outcomes with significant p values of 0.00 and 0.006, respectively. Chemotherapy or R.T., when used alone or when combined, had the worst O.S. and 5-year survival of 30.4%, 37.3%, or 23.3% respectively, and 9.3%, 12.8%, and 20.4%. Though MM was reported in 4.9% of NHB, the overall one-year survival was worst of all groups at 38.9%.

Another study from Zheng et al. published in 2020, elaborated on mucosal GI melanomas based on location of the primary and use of various treatment modalities [37].Our findings, concur with the study by Zheng et al. in terms of primary site of GI MM and survival [37]. However, their study [37]lacks effect of different ethnicities in detail and marital status on survival outcomes, which we have elaborated in our study.

Our study had limitations as discussed below. First, this is a retrospective analysis with associated limitations such as the available data. Second, the SEER database has an inclusion bias, with limited staging and metastatic data recorded. Third, the pathology details, including data on the resection margins are not available, which may again reflect on survival outcomes. Fourth, we have limited data on the type of surgery performed. We do not have information if the surgery had a curative versus palliative approach. Fifth, details about chemotherapy regimens used or radiation dosage used were not specified, nor was the intent of these therapies, adjuvant versus palliative approach was not defined. In addition, systemic treatment options for melanomas have evolved dramatically in the last decade compared to four decades ago, and details of the therapy become important when studying outcomes. Lastly, the SEERS database fails to mention about options provided by the physician, what the patient preferences or treatment options were based on the geographical availability with respect to treatment options.

Nonetheless, hope may be on the horizon with newer systemic adjuvant immunotherapeutic agents (temozolomide plus cisplatin) for resected MM, which has shown better over-all survival (p < 0.01) and relapse-free survival (p < 0.001), when compared to surgery alone or with surgery and high dose interferon IFN-α2b [25]. The rarity of the disease prolongs studies aimed at better outcomes. Most of the treatment strategies for chemotherapy have been extrapolated from cutaneous melanoma treatment, however with newer targeted systemic therapy including better understanding of the disease process with further identification and study at the molecular level (mutations in BRAF or KIT) of MM and advent of targeted immune check point inhibitors it has been estimated that the over-all survival of patients with MM should improve.

We feel that with availability of better chemotherapeutic agents and radiotherapy options, combined with surgery included into the therapeutic regimen in any form (S, SC, SRT, SCRT), the survival outcomes should improve in future for patients with gastrointestinal mucosal melanomas.

## Supplementary Information

**Additional file 1.** SEER data sheet on primary gastrointestinal melanoma.

## Data Availability

We have attached the data as a file with the submission.
